# Organoids as an Enabler of Precision Immuno-Oncology

**DOI:** 10.3390/cells12081165

**Published:** 2023-04-14

**Authors:** Junzhe Zhao, Antoinette Fong, See Voon Seow, Han Chong Toh

**Affiliations:** 1 Cancer and Stem Cell Biology Programme, Duke-NUS Medical School, Singapore 169857, Singapore; 2 Division of Medical Oncology, National Cancer Centre Singapore, Singapore 168583, Singapore; 3 Doctor of Medicine Programme, Duke-NUS Medical School, Singapore 169857, Singapore

**Keywords:** organoid, cancer, immunotherapy, tumour microenvironment

## Abstract

Since the dawn of the past century, landmark discoveries in cell-mediated immunity have led to a greater understanding of the innate and adaptive immune systems and revolutionised the treatment of countless diseases, including cancer. Today, precision immuno-oncology (I/O) involves not only targeting immune checkpoints that inhibit T-cell immunity but also harnessing immune cell therapies. The limited efficacy in some cancers results mainly from a complex tumour microenvironment (TME) that, in addition to adaptive immune cells, comprises innate myeloid and lymphoid cells, cancer-associated fibroblasts, and the tumour vasculature that contribute towards immune evasion. As the complexity of TME has called for more sophisticated human-based tumour models, organoids have allowed the dynamic study of spatiotemporal interactions between tumour cells and individual TME cell types. Here, we discuss how organoids can study the TME across cancers and how these features may improve precision I/O. We outline the approaches to preserve or recapitulate the TME in tumour organoids and discuss their potential, advantages, and limitations. We will discuss future directions of organoid research in understanding cancer immunology in-depth and identifying novel I/O targets and treatment strategies.

## 1. Introduction and Specifications

The past century witnessed a leap in our knowledge of how the immune system recognises, targets, and becomes tolerant of a neoplasm. Starting from the discovery of phagocytosis (Metchnikoff, 1908 Physiology or Medicine, together with Ehrlich), we have come to understand that monocytes and granulocytes can identify and kill cancer cells and also tame our self-defence. We observed the evasion of the foetus from the maternal immune system (Burnet and Medawar, 1960 Physiology or Medicine) and ushered in the field of immune tolerance. It has inspired many follow-on discoveries on immune checkpoints, regulatory lymphoid and myeloid cells, and the role of major histocompatibility complexes (MHC; Benacerraf, Dausset, and Snell, 1980 Physiology or Medicine), all of which play pivotal roles in the immunosuppression to and the tolerance of cancer. Landmark immunological milestones include how T cells differentiate self from non-self through the MHC complex (Doherty and Zinkernagel, 1996 Physiology or Medicine), as well as how phagocytes are activated (Beutler and Hoffmann, 2011 Physiology or Medicine) and antigens are presented (Steinman, 2011 Physiology or Medicine). These advances have contributed to our understanding of immune checkpoints (Allison and Honjo, 2018 Physiology or Medicine) and the development of humanised antibodies (Smith and Winter, 2018 Chemistry). Landmark phase 3 trials of immune checkpoint inhibitors (ICIs) proved the prolonged survival of many advanced cancer patients [1,2,3,4]. We realise that cancer is not simply a genetic disease where oncogenic driver mutations lead to uncontrolled proliferation but also a result of the complex interaction between malignant cells and the surrounding microenvironment. Without such interaction, cellular neoplasia may hardly become a full-blown tumour.

Mechanistic studies on the tumour microenvironment (TME) necessitate a system outside the human body that preserves or recapitulates what we observe in human cancers (even in this new era of tumour analysis at a single-cell resolution). This includes immune cells (lymphoid and myeloid), cancer-associated fibroblasts (CAFs), and endothelial cells, as well as their spatiotemporal organisation. The intricate nature of TME means developing translational pre-clinical TME models becomes one of the most daunting challenges in cancer immunotherapy [5]. Mouse models based on tumour cell seeding, genetic manipulation, or human tumour explants have offered great insight into cancer immunology and drug testing, yet they may not detail the evolution of tumourigenesis or the serial TME dynamics in humans. The 2D cancer cell lines offer narrower insight unless co-cultured with immune cells. Fortunately, we discovered the basement membrane matrix from the murine Engelbreth–Holm–Swarm tumour, which supports the 3D culture or even the self-assembly of human epithelial and stromal cells [6]. Organoid research has since offered great promise in immuno-oncology, yet there are still challenges to overcome.

Tumour organoids are ex vivo or in vitro 3D assemblies that recapitulate the spatiotemporal microanatomy and functions of the original tumour [7]. To study TME in an organoid system, specifications of the tumour organoids are twofold:

1. The organoids must retain all cell types of interest, including malignant and/or normal cells, stromal cells, and immune cells. The ratio and characteristics of these cells need to reflect those of the original tumour as well. Intuitively, the more cell types one includes, the more faithful the organoids are, compared to the in vivo tumour. However, the caveat is that it also leads to higher complexity and difficulty in studying detailed molecular and cellular mechanisms. While new technology, such as single-cell RNA sequencing (scRNA-seq) and multiplex immunofluorescence (mIF), have offered powerful tools to decipher tumour biology in higher dimensions, the bottom line of organoid modelling is that the minimum functional module for each research question is retained;

2. The organoids must be a 3D culture that mimics the structural and functional complexity of the original tumour. The extent to which such complexity is recapitulated depends very much on the study itself. In addition, tumours are heterogeneous in nature, as can be organoids. While organoids may recapitulate intra- and inter-tumour heterogeneity, it can lead to reduced reproducibility and robustness of the model itself [7]. As such, any conclusions drawn from organoids may have reduced statistical power. Here, the bottom line of organoid modelling is that for ex vivo organoids derived from patients, heterogeneity should be recapitulated by increasing patient recruitment and the locations where organoids are harvested; for in vitro organoids derived from consistent methods and cell sources, the results should be reproducible.

For instance, in the study of PD-1/PD-L1 inhibitors in hepatocellular carcinoma (HCC), organoids should retain at least the epithelial cells (malignant cells ± normal hepatocytes), T cells, and macrophages at physiologically relevant ratios. Macrophages must be in the system as they are important antigen-presenting cells (APCs) and the dominant PD-L1^+^ cells in HCC—often more than the tumour cells [8,9]. The distribution and characteristics of these cells need to mimic that of the actual HCC tumour tissue, and the degree of heterogeneity should reflect how these organoids are derived.

There are reviews comparing organoids with patient-derived xenografts (PDXs) in cancer research [10,11,12]. PDX can capture tumour–stromal interaction and closely mimic the clinical scenario when transplanted orthotopically. With a humanised immune system such as MISTRG [13] and HuMice [14], PDX can even capture complex tumour–immune interactions in vivo. However, orthotopic PDX models are labour-intensive and technically demanding [12]. In addition, there are differences in the pharmacokinetic profiles between humans and mice in drug testing [10]. Furthermore, the murine stroma, including CAFs, endothelial cells, and the extracellular matrix, possesses different properties from humans which are not entirely analogous (e.g., [15]). Finally, humanised mice may experience anaemia and limited adaptive immunity. Organoids, on the other hand, allow human-based investigation of specific interactions and high throughput drug screening. Recently, CRISPR-based organoid gene editing has also shown promise in disease modelling [16]. Different organoid approaches have also allowed varying degrees of model complexity and heterogeneity, whose advantages and limitations are reviewed in the following section. However, the ex vivo and in vitro models remain a simplification of the actual tumours, and characterising complex organoid models is challenging.

This review is not meant to be an exhaustive list of the past efforts in studying TME in organoids, which have been described [17,18,19,20,21,22,23]. Instead, by carrying forward discoveries from the Nobel laureates in this theme issue, we overview the methodology of TME studies in organoids, outline the new applications of organoids in immuno-oncology (I/O) research, and envision a future where organoid research benefits patients through improving precision I/O, such as by further elucidating complex resistance mechanisms. Organoids can be a potentially powerful platform informing a better understanding of cancer immunology and the cancer–TME interaction—building on immunological foundations first set forth by Nobel Laureates and other giants in immunology throughout history.

## 2. Approaches to TME Recapitulation or Preservation in Organoids

### 2.1. The Reductionist Approach: Matrigel-Based Organoids

Ex vivo tumour organoids refer to those derived directly from patient tissues, resembling a top-down approach. The sources of these tissues can be resected tumours or biopsies. The adjacent non-tumoural tissues are also obtained frequently during clinical procedures and are used to derive organoids for benchmarking. As discussed, the specification is to preserve the TME from the original tumour and, if possible, its heterogeneity.

Matrigel-based ex vivo organoids are generated by dissociating tissues into small cell clusters (often < 40 μm) or single cells, followed by Matrigel embedding. The culture media typically contains a growth factor cocktail to maintain the stem cell population. Patient-derived organoids (PDOs) for a variety of solid tumours have been generated, including breast [24], cervical [25], colorectal (CRC) [26], endometrial [27], gastric [28], glioblastoma [29], head and neck (HNSCC) [30], hepatobiliary [31,32], kidney [33], lung (NSCLC) [34], oesophageal [35], ovarian [36], pancreatic [37], prostate [38], sarcoma [39], and urothelial [40] cancers. These PDOs can be serially passaged and frozen, which makes long-term culture and bio-banking possible. They have also preserved the histology and genetic aberrations in the original tumour, which enables the testing of chemotherapy and targeted therapies. However, the culture condition seems to enrich only the cancer stem cells [41], with no characterisation of the stroma or TME in most studies. Spatial heterogeneity is also lost during PDO generation. Hence, co-culture with TME cell types is necessary to reconstitute the tumour-immune interaction, as discussed later in detail. Still, these PDOs have in fact provided a relatively clean platform for immune co-culture (TME reconstitution), which addresses specific immuno-oncology mechanisms. Intriguingly, microfluidic Matrigel embedding of low-volume tissues has recently yielded patient-derived micro-organospheres (MOS) that preserve the CRC TME [42,43], thanks to a rapid and automated pipeline for production and screening. Application for other cancer types is promising and awaits validation.

### 2.2. The Holistic Approach: PDOTS, ALI, TSC

While Matrigel-based PDOs aim to *reconstitute* TME by co-culture, the holistic approach *preserves* the original TME from patient tissues. Hence, the system comprises malignant, stromal, and immune components. Patient-derived organotypic tumour spheroids (PDOTS) [44] and air–liquid interface (ALI) [45] culture both involve tissue dissociation into larger fragments than Matrigel PDOs, followed by collagen gel embedding. Tissue slice cultures (TSCs) [46] directly embed vibratome-sliced tissues, often 200–300 μm thick and over 5 mm in diameter, in Transwell cell culture inserts. As with ex vivo Matrigel-based PDOs, non-tumoral tissues are processed along with tumours, while heterogeneity is preserved if possible.

**Figure 1:**
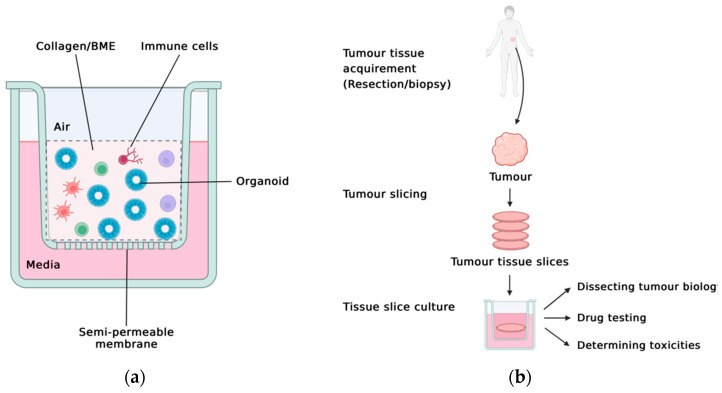
Schematics of air-liquid interface (ALI) culture and tissue slice culture (TSC) for ex vivo tumour: (**a**) ALI preserves the tumour-TME interaction with gentle tissue dissociation. BME: basement membrane extract. (**b**) TSC directly preserves sliced tissues as a whole. Figure created with BioRender.com.

PDOTS is derived from 40–100 μm large tissue fragments cultured in a microfluidic chip, with collagen as the matrix. In melanoma and Merkel cell carcinoma samples, PDOTS have been derived with lymphoid and myeloid fractions similar to the baseline tumour [44,47]. Air–liquid interface (ALI) culture has also demonstrated superiority over conventional Matrigel PDOs, which lack endogenous stroma. In ALI culture, tumours are minced (size unknown, surprisingly) and then sandwiched in extracellular matrix gels or collagen in the Transwell cell culture inserts. Media added in the outer plate can diffuse through the semi-permeable membrane to Transwell. The cells are directly exposed to oxygen, which increases the oxygen supply to the cells and preserves the organoids for 30–60 days (Figure 1a). ALI culture has enabled non-tumour and tumour-derived tissue samples to be cultured with native TME elements, circumventing the need for extensive supplementation of niche factors [48,49]. This culture system produces models that closely mimic in vivo airway epithelial cells in both cellular phenotype and transcriptional profiles [50,51]. A more recent study demonstrates that the propagated ALI culture accurately recapitulates the original tumour T cell receptor spectrum from patient tumour biopsies and accurately modelled tumour cytotoxicity with immune checkpoint blockade [45], allowing ex vivo modelling of TME interactions. However, ALI culture propagates mainly epithelial cells and leads to epithelial organoids, requiring a growth factor cocktail similar to Matrigel-based PDOs. The immune components in the culture can only be maintained for a short time, which limits the potential for long-term study of the TME and the epithelial–immune interactions [52,53,54].

Tissue slice cultures (TSCs) have been used to study tumour biology in intact samples (Figure 1b). In TSCs, solid tumour biopsies are embedded in agarose and sliced uniformly in sterile conditions before being plated in cell culture inserts or on hydrogels. As the stroma is fully retained, the maintenance is much simpler and cheaper than Matrigel PDOs, PDOTS, and ALI. Cells can be characterised via high dimensional flow cytometry, mIF, and scRNA-seq. In a recent study, tumour slices from CRC liver metastases are shown to preserve proliferation patterns and tumour–stromal morphology [46,55]. Alternatively termed patient-derived tumour explants/fragments, TSCs for brain [56], breast [57,58,59], kidney [59], lung [59,60], liver [61], melanoma [59], ovarian [59], pancreatic [62], prostate [63], and urothelial [64] cancers have all corroborated the findings and demonstrated the ability to retain molecular signalling and treatment responses [65,66]. TSCs hold promises in tailoring therapeutics and identifying tumour invasiveness. Yet, the complexity of the culture itself limits the long-term viability and robustness of the model. Tumour heterogeneity also necessitates the generation of multiple (often > 20) TSCs from one sample.

### 2.3. The Bottom-Up Approach: In Vitro Organoids

While ex vivo cultures have preserved or recapitulated the TME of many solid tumours, the success rate can vary considerably. For instance, the success rate is less than 30% for generating liver cancer PDOs [67]. Interestingly, the successful maintenance of tissues ex vivo does not seem to depend on tumour aetiology, such as receptor status for breast cancers [24] or viral versus non-viral aetiologies for HCC [67]. In addition, the TME is not always abundantly present in the tissues and is more challenging to maintain, which explains why most of the patient-derived organoid studies focus only on immune ‘hot’ tumours such as melanoma, NSCLC, and CRC. The question, then, is whether there is an organoid approach with improved viability and TME modelling for tumours with low ex vivo viability or little TME.

**Figure 2:**
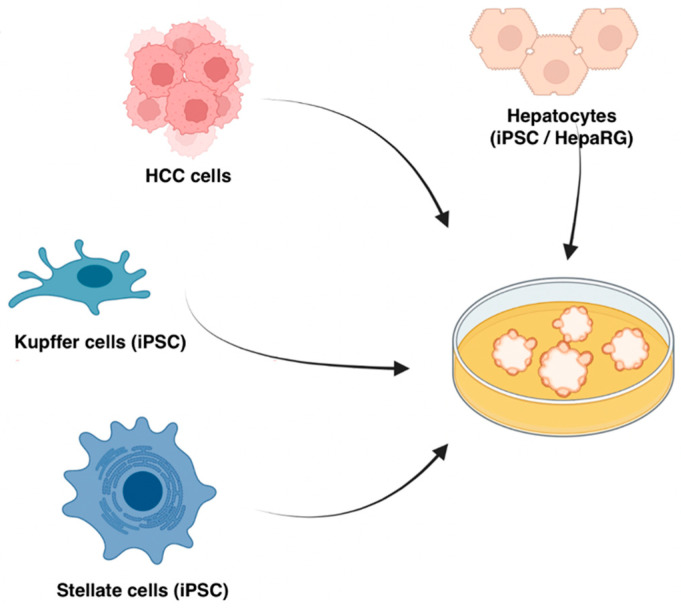
Schematic of the bottom-up approach for creating liver tumour organoids. Figure created with BioRender.com.

The solution to this question is perhaps to combine different cell types in vitro and generate organoids by Matrigel embedding, ultra-low attachment aggregation, and the hanging drop method. We can mix and match epithelial cells (iPSC-derived cells or cell lines), fibroblasts, endothelial cells, and immune cells to study the specific questions at hand. While patient-derived iPSCs may offer a fully autologous culture, allogenic iPSC organoids can also be used. To model the heterogeneous tumour tissues, normal parenchymal cells and tumour cell lines can be co-cultured, while the latter may be substituted by direct manipulation of the epithelial organoid to simulate tumorigenesis [68]. Patient-derived immune cells can then be co-cultured with these organoids to study tumour–immune interaction, though the alloreactivity needs to be controlled by either genetic manipulation or ensuring a low alloreactivity baseline. Our group has shown that HCC organoids can be generated by Matrigel embedding of iPSC-derived hepatocytes [69], Kupffer cells (KCs) [70], hepatic stellate cells (HSCs) [71], as well as HCC cell lines (unpublished, schematic in Figure 2). These cells self-assemble into organoids which can be treated with lipids to model non-alcoholic fatty liver disease (NAFLD)-related HCC.

A more sophisticated approach emerged in 2013 when Takebe and colleagues [72] created a 3D liver bud by co-culturing 3 cell types on a flat layer of Matrigel: iPSC hepatocyte-like cells, human mesenchymal stem cells (MSCs), and human umbilical vein endothelial cells (HUVECs). These self-assembled liver buds are vascularised and recapitulate most functions in human liver tissues. They subsequently showed that replacing the hepatocyte-like cells with other epithelial cell types leads to assembloids for various human organs and even cancer [73]. Such self-assembly processes are driven by MSCs, meaning that as long as the MSCs are functionally intact, tumour organoids can be generated by co-culturing tumour and TME cells with MSCs. It is noted, though, that MSCs may not be the main cell type in the original patient tumour, and they often interact directly with the immune cells [74], potentially confounding downstream investigation.

Finally, by carefully modulating the extracellular niche, multi-lineage differentiation from pluripotent stem cells such as iPSCs has offered a promising strategy that recapitulates the dynamics of the original tissue. Ouchi and colleagues [75] have differentiated organoids containing hepatocytes, KC-like cells and HSC-like cells from homogenous iPSC embryoid bodies (EBs). These liver organoids have been used to study NAFLD and drug-induced liver injuries [76], while the engraftment of HCC cells and other TME cells is also possible. Another example is the multi-lineage bone marrow organoids from iPSC EBs [77]. By culturing in a matrix mixture of Matrigel and type I and type IV collagen, these bone marrow organoids comprise myeloid, erythroid, and megakaryocyte lineages, which allow the rapid engraftment of patient-derived leukaemia and lymphoma cells. We recognise that alloreactivity concerns still exist for these organoids if T cells are to be incorporated into further studies. Thus, baseline establishment is vital.

## 3. Translational Immuno-Oncology Research with Organoids

### 3.1. Investigating T Cell and ICI Responses

Although unprecedented strides have been made in the role of ICIs across multiple cancers since Allison [78] first uncovered the immunoregulatory role of CTLA-4 and Honjo [79] of PD-1 in the 1990s, a significant proportion of cancer patients still do not benefit. Amongst the responders, intrinsic [80], adaptive, and acquired [81] resistance remains an ongoing challenge. Tumour organoids are excellent platforms for studying T cells and their response to ICIs. The co-culture of dissociated NSCLC/CRC PDOs with autologous T cells from peripheral blood mononuclear cells (PBMCs) enriches the tumour-specific population that, in turn, kills the tumouroids [82,83]. A recent study shows alloreactivity-depleted engineered T cells engage with breast cancer, HNSCC, and glioma PDOs in a manner associated with type I interferon signalling [84]. MOSs [43], PDOTS [44], ALI [45], and TSCs [46,59] have all recapitulated the anti-PD-1 response ex vivo, with the potential to test novel therapeutic combinations such as ICIs + CDK4/6 inhibitors [47]. In another study, PDOs were enriched with matched immune components and treated with pembrolizumab, ipilimumab, or nivolumab for seven days before viability assays [85], which may identify responders. Interestingly, CRC organoids show that the gut microbiota may affect ICI response [86], suggesting its role in immune regulation in addition to tumourigenesis, as examined in other studies [87,88] where organoids are infected with specific bacteria with no or low-dose antibiotics in the media. The pioneering Nobel discoveries of MHC and its restriction (1980, 1996) have laid the foundation for later findings on defective antigen presentation in cancer, which facilitate the evasion of T-cell-mediated killing. Indeed, in CRC [89] and breast cancer [90] organoids, drugs that stimulate MHC-I antigen presentation on the tumour cells can increase T cell cytotoxicity and ICI effectiveness.

Future translational research on tumour organoids and T cell/ICI response may focus on two key questions. First, can ex vivo organoids predict the response of neoadjuvant, adjuvant or upfront systemic ICI, based on tumour biopsies or resected tumours [91]? It is encouraging that there are multiple clinical trials on organoid-guided therapy—such as NCT04777604 for the neoadjuvant space, NCT04736043 for the adjuvant space, and even NCT04931381 in advanced inoperable cancer (Supplementary Table S1 has a complete list)—all of which have offered great promise in validating organoid-guided I/O. Matrigel-based organoids may not serve this purpose well since the cultures may take weeks to stabilise, while PDOTS, ALI, and TSC may establish viable, TME-preserving cultures rapidly. Second, can organoids capture the spatiotemporal dynamics of checkpoint expression [9] and the changes ICI causes in the tumour cells, the TME, and the adjacent normal? This will offer insight into the characteristics of responders versus non-responders and the tumour evolution towards ICI resistance.

### 3.2. Unravelling the Functions of TME Cells

Metchnikoff might not have foreseen a century ago [92] that different macrophage populations possess distinct influences on cancers beyond phagocytosis, either in reducing the efficacy of therapies or limiting tumour growth in general. In 2004, Alberto Mantovani and colleagues proposed an M1–M2 spectrum of macrophage polarisation [93], and they subsequently defined the concept of tumour-associated macrophages (TAMs) [94]. Ovarian cancer organoids have shown that UBR5 from tumour cells drives the recruitment and immunosuppressive reprogramming of TAMs [95]. In intestinal adenoma, co-cultured macrophages promote the growth of organoids in a prostaglandin E2-dependent manner, while macrophages themselves adopt a TAM-like phenotype [96]. This reciprocal interaction is abrogated by celecoxib, a selective COX-2 inhibitor. Another co-culture of pancreatic cancer PDOs, U937 monocytes, and primary pancreatic stellate cells leads to increased PDO invasiveness and an M2-like phenotype of U937 [97], whose depletion significantly increases PDO sensitivity to chemotherapy. Finally, autologous co-culture of CRC PDOs with PBMC-derived CD8^+^ cytotoxic T lymphocytes (CTLs) and macrophages shows that a high sirtuin 1 (SIRT1) level in CRC cells increases macrophage infiltration and M2 polarisation, contributing to CTL dysfunction [98]. While tumour organoids have offered insight into how TAMs may act as friend or foe, caution must be taken when optimising the culture conditions (especially the external growth factor/cytokine supply), as they might shift the phenotypes of many cell types and mask the true effect of tumour–immune interaction. Future organoid-based TAM research may detail how TAMs cause T cell dysfunction [99], tumour cell inflammation, metabolic derangements [100], and increased angiogenesis [101,102] (discussed below). In fact, an M1–M2 dichotomy may be an oversimplification for TAMs in vivo, which can possess markers for both M1 and M2 at the same time [103]. We know that macrophages are pro-inflammatory during pre-tumour necroinflammation, which are subsequently polarised towards immunosuppressive cells in the tumour. However, what leads to this cascade of TAM reprogramming is not understood; thus, organoids may offer insight into this profound question.

Many years after Burnet and Medawar’s discovery of the concept and principles of immune tolerance, myeloid-derived suppressor cells (MDSCs) were described as a vital contributor to cancer immunosuppression. Several studies have shown viable MDSCs in PDOs, whose depletion sensitises ICI response [104,105]. However, detailed, longitudinal examination of MDSCs in organoids remains rare. The same holds true for dendritic cells (DCs) [106], neutrophils, and other granulocytes [107]—important TME cell types yet to be further studied in organoids.

While lymphoid organoids have been successfully generated in vitro [108,109], the recapitulation or study of B cells in tumour organoids remains challenging and hence very limited [45]. As more research has uncovered the multifarious roles of B cells in the TME [110], further work on B-cell-containing tumour organoids may be necessary.

Endothelial cells (ECs) are another critical cell type in the TME. While it is well known that angiogenesis, a hallmark of cancer [111], can be driven by tumour cells rather aberrantly [101,112,113], we now understand that the endothelium does much more than the sheer lining of blood vessels. For instance, ECs can mediate metastasis [112,114], present antigens via MHC-II [115], reprogram the myeloid phenotypes [116,117], and regulate tissue fibrosis [118]. Tumour organoids have uncovered new roles of ECs other than vascularisation. ECs utilise the tumour cell secretome and hijack the M2-like TAM polarisation for angiogenesis in HCC [101] and glioblastoma [102] organoids. Through their angiocrine functions, ECs in tumour organoids have been shown to drive a pro-inflammatory environment and M1-like TAM polarisation, antagonising the effect of M2-like TAMs [101]. Organoids have also revealed the importance of angiocrine signalling in the survival of pancreatic cancer cells [119,120] and in promoting ovarian cancer metastasis [121]. Strikingly, ovarian cancer organoids with ECs bear a close resemblance to metastatic tumour spheres in the peritoneum or ascites of patients [121,122]. Careful consideration is needed if HUVECs are used in organoids, as they may behave differently from tumour ECs and tissue-specific ECs, such as liver sinusoidal endothelial cells (LSECs) [123]. Further organoid studies are warranted on the spatiotemporal dynamics between ECs, TAMs and T cells, the vascular normalisation by anti-angiogenic drugs [124,125], and the effect of ECs in pre-cancer [126].

Tumour organoids provide the most physiologically relevant platform ex vivo and in vitro for studying CAFs. Defined as the tumour mesenchymal cells that do not bear epithelial, immune, or endothelial markers [127], CAFs in pancreatic cancer maintain their phenotypes only in organoids but not in mono-culture [120,128]. The distinction between α-SMA-positive myofibroblastic CAFs (myCAFs) and IL-6-positive inflammatory CAFs (iCAFs) [128] is driven by TGF-β and IL-1 signalling, respectively [129]. CAF plasticity in pancreatic cancer leads to distinct TME of a reactive, immune-hot subtype versus a deserted, immune-cold counterpart [130]. Intriguingly, while the immune-reactive TME CAFs promote tumour organoid growth and are associated with lower disease-free survival, the immune-deserted TME CAFs confer chemoprotection to the organoids and are associated with lower overall survival [130]. Additionally, tumour organoids for CRC [131,132], HCC [133], and prostate cancer [134] have all shown a similar trophic effect of CAFs and the conferral of therapeutic resistance, seemingly relying on paracrine signalling. It is noted that patient-derived CAFs rapidly differentiate towards myCAFs in vitro, and thus protocol optimisation is key in preserving the CAF phenotype in organoids [132]. Investigation into the recruitment and reprogramming of CAFs, and their paracrine effect on tumour cells and immune cells, will yield exciting insight into the immunoregulatory functions of CAFs and offer new therapeutic opportunities, which are discussed next.

### 3.3. Testing of Novel Precision Immuno-Oncology Strategies

**Figure 3:**
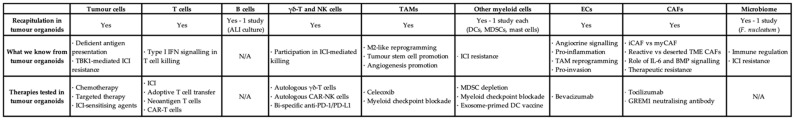
Tabulated summary of different cell types in tumour organoids, the knowledge added, and the therapeutics tested. TAM: tumour-associated macrophage; EC: endothelial cell; CAF: cancer-associated fibroblast; ALI: air–liquid interface; DC: dendritic cell; MDSC: myeloid-derived suppressor cell; ICI: immune checkpoint inhibitor; IFN: interferon; iCAF: inflammatory CAF; myCAF: myofibroblastic CAF; TME: tumour microenvironment; CAR: chimeric antigen receptor.

The past decade has witnessed the therapeutic revolution ICI has brought to the lives of many cancer patients simply by unleashing the brakes on effector T cells. Now, the reality of tumour heterogeneity, the advance of our knowledge in the TME, and the breakthroughs of our ability to interrogate tumour biology have all called for the advent of personalised I/O. Organoids are, and will continue to be, a key driver of precision I/O with their superior accessibility, rapid throughout, and good robustness [135,136]. We begin by summarising in Figure 3 what we have discovered about different cell types from tumour organoids and whether therapies targeting each cell type have been tested on them, discussed in detail next.

With combination immunotherapies proving superior survival outcomes across more and more cancers and even in the adjuvant setting [137], and with rising numbers of ongoing combination trials awaiting definitive results, combining ICIs and other therapeutic modalities will define a significant space in cancer immunotherapy strategies in the coming years [138]. New targets on the tumour cells have emerged that directly influence ICI-mediated cytotoxicity. PPARγ-agonists, such as pioglitazone, reprogramme the metabolism and inflammatory response of microsatellite-stable (MSS) CRC cells and increase their PD-L1 expression, sensitising these seemingly resistant organoids to ICI [139]. Targeting TBK1, a kinase with multifaceted functions in innate immunity and cell proliferation, also enhances ICI response, as seen in PDOTS of multiple cancers [140].

Combining ICIs with TME-targeting therapies may also lead to improved clinical response [141], and some of these strategies have been tested in tumour organoids. As discussed earlier, celecoxib in tumour organoids can abrogate the TAM-tumour stem cell interaction [96]. Celecoxib or other NSAIDs (e.g., aspirin in NCT00565708) may be attractive in targeting TAMs, yet their overall clinical effect remains uncertain. Depletion of MDSCs in gastric cancer PDOs increases anti-PD-1/PD-L1-induced killing [104], suggesting the immunosuppressive role of myeloid checkpoints besides PD-L1, such as arginase-1 [142] and VISTA [143,144]. Clinical trials are underway to test anti-VISTA monotherapy or combination with anti-PD-1 in advanced solid tumours (e.g., NCT05082610) [145]. Surprisingly, histamine, via H1 receptors on TAMs, confers ICI resistance by polarising TAMs towards M2 with increased VISTA expression [146]. Organoid testing for H1-antihistamines, anti-VISTA, and other myeloid checkpoint inhibitors may offer a rapid prediction, in-depth molecular insight, and longitudinal monitoring. In addition, ICI combined with bevacizumab has already shown benefits in phase 3 trials [137,147], which warrant organoid studies of EC-targeting therapies [124]. Furthermore, small molecule inhibitors or blocking antibodies targeting FGFR or TGF-β receptors may interfere with CAF activation and action [127], as seen in organoids [148]. However, their clinical benefit remains uncertain [149], suggesting the robustness of CAFs and the difficulty in targeting them. Intercepting the IL-6 axis may also abrogate tumour–CAF interaction [150], yet the effect of drugs (e.g., tocilizumab) both on the TME and systemically is concerning. Crucially, culture conditions, such as growth factor supply and hypoxia, may directly influence the readouts of organoid testing, as discussed in earlier sections.

Adoptive cell transfer of tumour infiltrating lymphocytes (TILs) or tumour-reactive T cells, especially neoantigen-specific T cells [151,152], has emerged as a promising strategy of precision I/O [153]. Lymphodepletion with fludarabine/cyclophosphamide or similar non-ablative conditioning regimens creates a favourable environment for the engraftment, survival, and optimal in vivo expansion of the infused therapeutic T cell product [153,154]. ICIs may be synergistic or additive to adoptive cell therapy [155]. Organoids can boost T cell therapy translation by rapidly enriching tumour-reactive T cells [82,83], unravelling the regulation [156,157] and heterogeneity [158] of MHC-I neoantigen presentation and, crucially, providing a platform for neoantigen validation [155] and autologous killing assays [43,159].

Chimeric antigen receptor (CAR)-T cells have shown unequivocal clinical efficacy against B cell malignancies and multiple myeloma. Testing against solid tumours has shown some encouraging clinical efficacy, although significant challenges from the immunosuppressive TME still exist [160]. The delicate design of the CAR-T cell construct and its activation mechanisms leads to their potent killing capacity and sustained presence and anti-tumour activity [161,162,163]. Organoid-killing assays have shown success in CAR-EGFRvIII T cells for glioblastoma [29], CAR-HBsAg for HBV-HCC [164], as well as CAR-CD70 for renal cell carcinoma [165]. Further investigation on organoid-CAR-T co-culture may offer solutions to CAR-T cell exhaustion in vivo and discover novel targets tackling tumour heterogeneity. For instance, the efficacy of CAR-T cells might be limited by the physical barrier of a fibrotic tumour stroma [166]. Organoid modelling of tissue fibrosis has shown the deposition of collagen and other extracellular matrix proteins [75,167]. By modifying the mechanical and biochemical properties of the matrix or hydrogels in organoid culture, we may unravel the spatiotemporal dynamics of CAR-T infiltration into and their killing of tumouroids.

Finally, NK and γδ-T cell therapies, both autologous and allogenic, have received greater attention now due to their potent anti-tumour capabilities. NK cells typically react to stress ligands via interactions including NKG2A–HLA-E and NKG2D–MIC-A/B [168], while γδ-T cells recognise phosphoantigens, such as exogenous zoledronic acid (ZOL), to exert cytotoxicity [169]. Strikingly, a recent study with ovarian cancer organoids shows that bi-specific PD-1/PD-L1 antibody activates NK cells in addition to CTLs [170], while another study with CRC organoids demonstrates the anti-tumour effect of ICIs from γδ-T cells (rather than αβ-T) when there is MHC-I defect on tumour cells [171], underlining their importance in I/O effectiveness. Organoid testing has also shown tumoricidal activity of γδ-T cells [172] and (CAR-)NK cells [173] for melanoma and mesothelioma, respectively. Importantly, while 3D killing assays have been performed and published extensively for NK and γδ-T cells, most “tumours” are only spheroid aggregates of cancer cell lines, which do not recapitulate the TME. Killing assays on organoids, on the other hand, will unveil distinct mechanisms that facilitate or inhibit cell therapies, many of which arise from interactions with the TME rather than with tumour cells alone. For instance, our group is studying the distinct phenotypes between NKG2A(+) and NKG2A(-) γδ-T cells, where tumour organoid co-culture may offer a better understanding.

## 4. Concluding Remarks

Advances in basic, translational, and clinical immunology, coupled with an ever-increasing repertoire of technological know-how, have greatly empowered the immune system to be harnessed even more effectively against cancer. All such benefit to the lives of cancer patients is rooted in the foundational, pioneering discoveries of innate and adaptive immunity by the Nobel laureates and many others contributing to the field. We celebrate their inspirational work, and we also applaud the other great discoveries in T cell education and maturation [174], TCR [175], myeloid plasticity [176], inflammation and immunity [177], and adoptive cell therapy [153,160,162,178]. Amongst different cancer models, organoids, with the recapitulation or preservation of the TME, sit at the delicate balance of human-based biology and accessible experimentation, offering powerful insight into tumour–immune interactions—“A semblance great, the shadow of a shade” (Tao Te Ching). As animal studies are no longer mandatory for all new drug approvals by the FDA [179], tumour organoids will assume an even more pivotal role in guiding patient management and therapeutic development. By refining ex vivo and in vitro organoid modelling and detailing the spatiotemporal dynamics of the TME, we can potentially improve precision I/O and examine even deeper into the real-time biology, the evolution of immune cells, and how they intimately interact with cancer and its surroundings.

## Data Availability

Not applicable.

## Supplementary Materials

The following supporting information can be downloaded at: https://www.mdpi.com/article/10.3390/cells12081165/s1. Table S1: Summary of clinical trials on tumour organoid-guided therapy at ClinicalTrials.gov (accessed on 30 January 2023). Only trials with the status Recruiting, Not yet recruiting, Active not recruiting or Completed are included.
